# Evidence of the Berezinskii-Kosterlitz-Thouless phase in a frustrated magnet

**DOI:** 10.1038/s41467-020-19380-x

**Published:** 2020-11-06

**Authors:** Ze Hu, Zhen Ma, Yuan-Da Liao, Han Li, Chunsheng Ma, Yi Cui, Yanyan Shangguan, Zhentao Huang, Yang Qi, Wei Li, Zi Yang Meng, Jinsheng Wen, Weiqiang Yu

**Affiliations:** 1 Department of Physics and Beijing Key Laboratory of Opto-electronic Functional Materials and Micro-nano Devices, Renmin University of China, Beijing, 100872 China; 2 National Laboratory of Solid State Microstructures and Department of Physics, Nanjing University, Nanjing, 210093 China; 3 Institute for Advanced Materials, Hubei Normal University, Huangshi, 435002 China; 4 Beijing National Laboratory for Condensed Matter Physics and Institute of Physics, Chinese Academy of Sciences, Beijing, 100190 China; 5 School of Physical Sciences, University of Chinese Academy of Sciences, Beijing, 100190 China; 6 School of Physics, Key Laboratory of Micro-Nano Measurement-Manipulation and Physics (Ministry of Education), Beihang University, Beijing, 100191 China; 7 Center for Field Theory and Particle Physics, Department of Physics, Fudan University, Shanghai, 200433 China; 8 State Key Laboratory of Surface Physics, Fudan University, Shanghai, 200433 China; 9 Collaborative Innovation Center of Advanced Microstructures, Nanjing University, Nanjing, 210093 China; 10 International Research Institute of Multidisciplinary Science, Beihang University, Beijing, 100191 China; 11 Department of Physics and HKU-UCAS Joint Institute of Theoretical and Computational Physics, The University of Hong Kong, Pokfulam Road, Hong Kong, SAR China; 12 Songshan Lake Materials Laboratory, Dongguan, Guangdong 523808 China

**Keywords:** Magnetic properties and materials, Phase transitions and critical phenomena, Topological insulators

## Abstract

The Berezinskii-Kosterlitz-Thouless (BKT) mechanism, building upon proliferation of topological defects in 2D systems, is the first example of phase transition beyond the Landau-Ginzburg paradigm of symmetry breaking. Such a topological phase transition has long been sought yet undiscovered directly in magnetic materials. Here, we pin down two transitions that bound a BKT phase in an ideal 2D frustrated magnet TmMgGaO_4_, via nuclear magnetic resonance under in-plane magnetic fields, which do not disturb the low-energy electronic states and allow BKT fluctuations to be detected sensitively. Moreover, by applying out-of-plane fields, we find a critical scaling behavior of the magnetic susceptibility expected for the BKT transition. The experimental findings can be explained by quantum Monte Carlo simulations applied on an accurate triangular-lattice Ising model of the compound which hosts a BKT phase. These results provide a concrete example for the BKT phase and offer an ideal platform for future investigations on the BKT physics in magnetic materials.

## Abstract (web summary)

The Berezinskii-Kosterlitz-Thouless (BKT) phase transition has long been sought yet undiscovered directly in magnetic materials. Here, the authors identify two phase transitions with BKT fluctuations detected by NMR and critical scaling behavior in magnetic susceptibility expected for the BKT transition in a frustrated magnet TmMgGaO4.

## Introduction

Topology plays an increasingly important role in understanding different phases and phase transitions in correlated quantum matters and materials. One prominent example is the Berezinskii–Kosterlitz–Thouless (BKT) mechanism in two-dimensional (2D) systems^1–5^, which is associated with the binding and unbinding of topological defects. The BKT transition cannot be characterized by conventional order parameters and constitutes the earliest example of phase transition beyond the Landau–Ginzburg paradigm of spontaneous symmetry breaking. Historically, the BKT mechanism was introduced in the *XY* spin model and long predicted to occur in magnetic thin films^1,4^. In experiments, signatures of the BKT transition have been observed in superfluid helium films^6^, as well as in 2D superconducting films^7,8^ and arrays^9^. Regarding the original proposal in layered *XY*-type magnets, despite intensive efforts^10–15^, direct and unambiguous observation of the BKT transition is still lacking. One major obstacle is the three-dimensional (3D) couplings in the magnets, although weak, will inevitably enhance the confining potential of vortices^15^, leading to 3D ordering that masks the BKT transition. Therefore, it is of fundamental interest to find and identify BKT materials that could overcome the obstacle and study the topology-related low-energy dynamics.

Recently, a layered frustrated rare-earth antiferromagnet TmMgGaO_4_^16–18^ was reported to ideally realize the triangular-lattice quantum Ising (TLI) model^19^. The relatively large interlayer distance of 8.3774 Å along the *c* axis gives rise to excellent two dimensionality^17^ and no sign of conventional 3D phase transition was observed in either specific heat or magnetic susceptibility measurements. Nevertheless, it was reported from neutron scattering that TmMgGaO_4_ ordered below  ~1 K into an antiferromagnetic phase with a sixfold symmetry breaking^16,18^, which closely resembles the ground state of the TLI model originated from an order-by-disorder mechanism^20,21^. At higher temperatures, the effective *XY* degrees of freedom emerge and the BKT mechanism is expected to come into play^21^.

In TmMgGaO_4_, each Tm^3+^, with a 4*f*^12^ electron configuration and a spin–orbit moment *J* = 6, forms a non-Kramers doublet due to the crystal-electric-field splitting. The doublet is well separated from the rest levels by about 400 K^16^ and can thus be regarded as an effective spin-1/2. There is further a fine energy splitting within the doublet, induced by the local trigonal crystal field^17^, acting as an intrinsic “transverse field” applied on the effective spin. From the magnetization measurements^16–18^, one observes that Tm^3+^ ions contribute highly anisotropic Ising-type moments with *J*_*z*_ = ±6 along the *c* axis, resulting in an effective out-of-plane *g*-factor ~ 13.2^16,19^. On the contrary, the effective in-plane *g*-factor and dipolar moment in the *ab* plane are negligible.

Facilitated by this feature in TmMgGaO_4_, in this work we employed nuclear magnetic resonance (NMR), a sensitive low-energy probe, to detect the BKT phase. We applied a moderate in-plane field of 3 T, which is adequate to collect the ^69^Ga NMR signals and, at the same time, hardly disturbs the low-energy electronic states of the material. This is important, as in the TLI model that is believed to accurately model TmMgGaO_4_^19^, the BKT phase can be fragile against out-of-plane fields^22–24^, thus posing a challenge to NMR measurements. Taking advantage of the fact that in-plane moment in TmMgGaO_4_ is mostly multipolar^16,19^, our NMR experiments with in-plane fields, which merely couple to the nuclear spins, can clearly identify the BKT phase in the material.

**Fig. 1:**
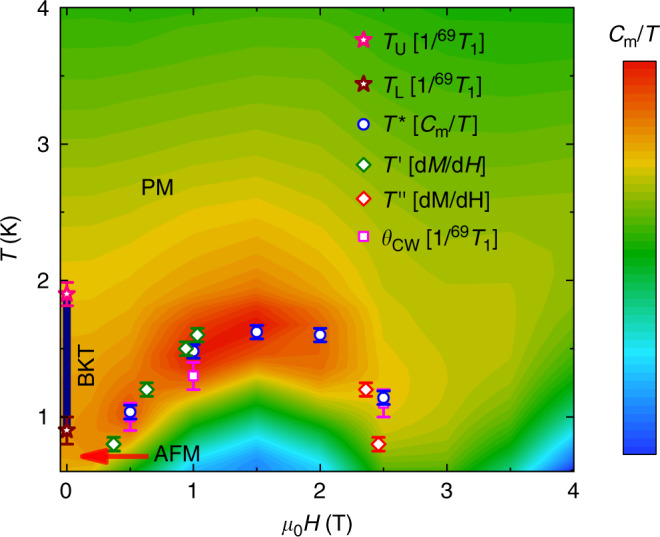
Phase diagram of TmMgGaO_4_ under out-of-plane magnetic fields. Under zero field, there are paramagnetic (PM), BKT, and antiferromagnetic (AFM) phases. The *T*_U_ (*T*_L_) is the upper (lower) BKT transition temperature, determined from the plateau structure in the NMR spin-lattice relaxation rate 1/^69^*T*_1_ (see Fig. 2**c** for details). The BKT phase between *T*_U_ and *T*_L_ is illustrated by the solid vertical line, while the AFM regime is indicated by the arrow. The contour background depicts the magnetic specific heat *C*_m_/*T* at various fields and temperatures, with data adapted from ref. ^16^ and plotted in logarithmic scale. T^*^ corresponds to the maximum of *C*_m_/*T* at each field, signifying the position of strong magnetic fluctuations. $T^{\prime}$(*T*″) denotes the temperature at a specific field where a peak is found in the differential susceptibility *dM*/*dH*, shown in Fig. 3b. The Curie–Weiss temperature *θ*_CW_ is obtained from the 1/^69^*T*_1_*T* (see Supplementary Fig. 1). Remarkably, T^*^, $T^{\prime}$, *T*″, and *θ*_CW_ all collapse to the same phase boundary between the BKT-like regime and AFM phase. A magnetically ordered phase is supposed to lie below the dome-like boundary. Errors represent 1 SD throughout the paper.

As shown in Fig. 1, from our NMR measurements of the spin-lattice relaxation rate 1/*T*_1_, we identify *T*_U_ ≃ 1.9 K and *T*_L_ ≃ 0.9 K, which represent the upper- and lower-BKT transitions, where a critical BKT phase resides at zero magnetic field in between the high-*T* paramagnetic and low-*T* antiferromagnetic phases. This finding is further substantiated by our scaling analysis of the measured susceptibility data near *T*_L_, as well as the simulated NMR and susceptibility data using large-scale quantum Monte Carlo (QMC) calculations.

## Results

### NMR probe of the BKT phase

**Fig. 2:**
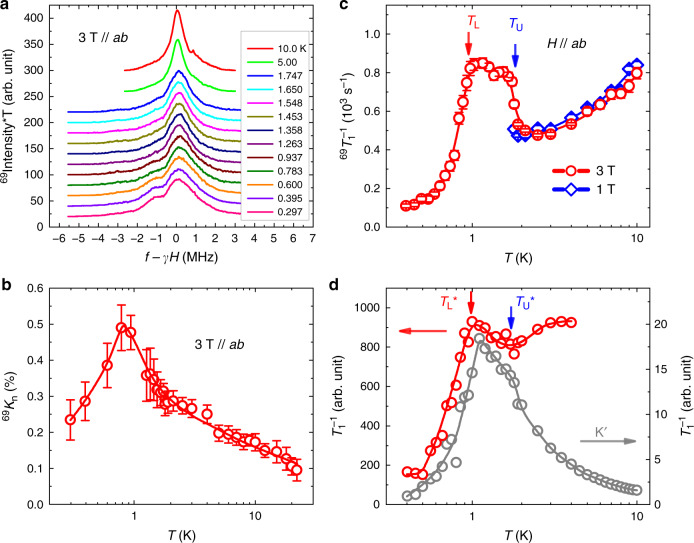
NMR spectra and spin-lattice relaxation rates of TmMgGaO_4_. **a** ^69^Ga NMR spectra at different temperatures under a 3 T in-plane field, with zero frequency corresponding to *γH* = 30.692 MHz. Data are shifted vertically for clarity. At high temperatures, the spectra are roughly symmetric, whereas for *T* ≤ 2 K, a shoulder-like structure can be resolved on the left side of the main peak. **b** NMR hyperfine shift ${}^{69}{K}_{n}=(\bar{f}/\gamma H-1)\times 100 \%$ as a function of temperature, where $\bar{f}$ is the average frequency of each spectrum. **c** NMR spin-lattice relaxation rate 1/^69^*T*_1_ vs. temperature measured under in-plane fields of 3 T and 1 T. A plateau-like feature, characterizing strong magnetic fluctuations, is observed between *T*_L_ ≃ 0.9 K (lower-BKT transition) and *T*_U_ ≃ 1.9 K (upper BKT transition). **d** 1/*T*_1_ data computed from the dynamical spin–spin correlation function with contributions from all momentum points [c.f., Eq. (3) and see the hyperfine form factor in Supplementary Note 2] in the Brillouin zone (left scale) and from *K*′ point in the vicinity of the *K* point (right scale), through large-scale QMC simulations (see “Methods”).

The obtained NMR spectra with an in-plane magnetic field *μ*_0_*H* = 3 T are shown in Fig. 2a at representative temperatures. To better resolve the magnetic transition, the hyperfine shifts ^69^*K*_n_ of the spectra were analyzed and plotted in Fig. 2b as a function of temperature. Upon cooling, ^69^*K*_n_ peaks at about 0.8–0.95 K and then starts to drop at lower temperatures. Therefore, the ordering temperature is determined to be *T*_L_ ≃ 0.9 K, consistent with neutron scattering experiments^16,18^. In addition, both the second moments (width of the NMR spectra) and the third moments (asymmetry of the spectra) of the spectra change dramatically below  ~ 2 K, suggesting the onset of local hyperfine fields enhanced by the static or quasi-static magnetic ordering (Supplementary Fig. 4). These two characteristic temperatures signal the two-step melting of magnetic order through two BKT transitions, suggesting an intermediate floating BKT phase in the system. We suspect that there is some inhomogeneity of the local hyperfine fields, which is very likely caused by the quenched disorder from Mg/Ga site mixing^16^, although no significant influence on the electronic and more importantly the magnetic properties is seen (see more detailed discussions in Supplementary Note 4).

The spin-lattice relaxation rate 1/*T*_1_ provides a highly sensitive detection of low-energy spin fluctuations^25–29^, and thus the BKT transition. In Fig. 2c, we show the 1/^69^*T*_1_ obtained under in-plane fields *μ*_0_*H* = 3 T and 1 T, which reflects intrinsic spin fluctuations with zero out-of-plane field. At 3 T, 1/^69^*T*_1_ first decreases upon cooling from 10 K then suddenly increases below *T*_U_ ≈ 1.9 K, indicating the onset of strong low-energy spin fluctuations. The data at 1 T show similar behaviors. Below *T*_L_ ≃ 0.9 K, 1/^69^*T*_1_ drops sharply, consistent with the onset of the magnetic ordering as also inferred from the hyperfine shift. Here, 1/^69^*T*_1_ is dominated by the gapped long wavelength excitations in the ordered state. At the magnetic phase transition, a peaked feature in 1/*T*_1_ develops, caused by the gapless low-energy spin fluctuations with diverging correlation length. Remarkably, at temperatures between *T*_U_ ≃ 1.9 K and *T*_L_ ≃ 0.9 K, 1/^69^*T*_1_ exhibits a plateau-like structure, indicating the emergence of a highly fluctuating phase with diverging spin correlations yet no true long-range order, which is the hallmark of a BKT phase^1–5^. Therefore, it is for the first time that such a phase is unambiguously observed in a magnetic crystalline material.

Our unbiased QMC simulations on the TLI model of the material (see “Methods”), with accurate coupling parameters determined in ref. ^19^, quantitatively justifies the existence of the BKT phase between *T*_L_ and *T*_U_. We computed 1/*T*_1_ and compared with the experiment below. Figure 2d shows the calculated 1/*T*_1_ by including fluctuations from all momentum points in the Brillouin zone (cf. Supplementary Fig. 2) and compare to that from *K*′ (around the *K* point at the corner of Brillouin zone). The former shows a decrease upon cooling below 4 K and then an upturn above ${T}_{{\rm{U}}}^{* }\simeq 2$ K, followed by a rapid decrease below ${T}_{{\rm{L}}}^{* }\simeq 1$ K. These behaviors are in excellent agreement with the measured 1/^69^*T*_1_. The latter reflects gapless excitations of the *XY* degrees of freedom emergent in the BKT phase, where the calculated 1/*T*_1_ from *K*′ exhibits an anomalous increase down to ${T}_{{\rm{U}}}^{* }$, below which the increment slows down. The contribution to 1/*T*_1_ near the *K* point reaches a maximum at ${T}_{{\rm{L}}}^{* }$ and drops rapidly below it. The absence of critical spin fluctuations at momentum away from the K point suggests that the measured 1/^69^*T*_1_ below 2 K is mainly contributed by excitations around the *K* point (see Supplementary Note 3).

Overall, the quasi-plateau behaviors in the QMC results and the two characteristic temperature scales are in full consistency with the NMR measurements. This constitutes both strong support for the accurate quantum many-body modeling of the material TmMgGaO_4_ and also solid proof of the BKT phase therein detected by NMR. Nevertheless, we note that there are still subtle differences between the experimental and numerical data. Needless to say, the real material is always more complicated than our theoretical minimal model. For example, influences from higher crystal-electric-field levels above the non-Kramers doublet, the interlayer couplings not included in our model calculations, and the lack of knowledge on the precise local hyperfine coupling constant, etc., may explain the difference remaining between Fig. 2c, d.

### Universal magnetic susceptibility scaling

**Fig. 3:**
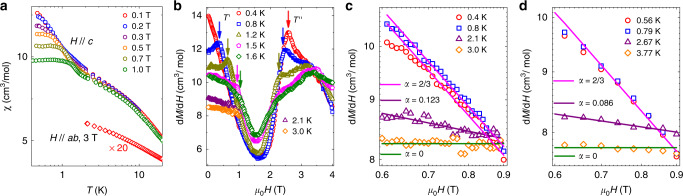
Uniform magnetic susceptibility of TmMgGaO_4_ and scaling analysis. **a** dc susceptibility *χ* as functions of temperatures under small out-of-plane (*H*//*c*) and in-plane (*H*//*ab*) fields. The latter is multiplied by a factor of 20 for visualizing purpose. The deviation of data below 2 K indicates the entry to the BKT phase and the field-suppression of magnetic correlations. **b** The differential susceptibility *dM*/*dH* under out-of-plane fields at different temperatures. The kinks at low fields, as denoted by the down arrows, suggest the transition from the BKT-like phase to the ordered phase (under the dome in Fig. 1) with increasing fields. The peaked features at high fields suggests a quantum phase transition to the polarized phase. **c** Fits of *dM*/*dH* to the power-law scaling function *dM*/*dH* ~ *H*^−*α*^ with *α* = 2/3 for the 0.4 and 0.8 K data, and *α* = 0.123 for 2.1 K data in the log-log scale. The 3 K data follow the *α* = 0 line in the paramagnetic phase. **d ***dM*/*dH* by the QMC calculations in the same field and temperature range as in **c**, and fits to the power-law function with exponents *α*, which give consistent results as experiments.

Magnetic susceptibility *χ* measurements were also performed to strengthen the finding of the BKT phase. In Fig. 3a, we show the overall temperature dependence of *χ* at different out-of-plane fields. For *T* ≳ 2 K, *χ* increases monotonically upon cooling and barely changes with fields. However, for *T* ≲ 2 K, approximately the upper BKT transition *T*_U_ as obtained from the 1/^69^*T*_1_ measurements, *χ* increases as the field decreases, suggesting the onset of peculiar magnetic correlations. With further cooling, the susceptibility gets flattened with temperature. The magnetization *M*(*H*) was further measured at selected temperatures (data shown in Supplementary Fig. 6), and for the sake of clarity, the differential susceptibility *dM*/*dH* is plotted in Fig. 3b. At around 2.5 T, a pronounced peak can be observed at low temperature, indicating the existence of a quantum phase transition, and the phase at lower fields should be a magnetically ordered phase, although its precise nature remains to be uncovered. Besides the high-field feature, for temperatures at 0.8 K and above, a kinked feature is clearly resolved on each *dM*/*dH* curve at low fields, whereas at 0.4 K, the low-field kink disappears, which posts a question of whether there is a quantum transition or merely a crossover from the zero-field AFM phase to the finite-field ordered phase under the dome in Fig. 1. The temperature and field values indicated by the down arrows in Fig. 3 are denoted as $T^{\prime}$ and *T*″ in the phase diagram (Fig. 1).

Field-theoretical analysis of the TLI model^23,24^ has predicted that upon applying a small out-of-plane field, the differential susceptibility *dM*/*dH* shall exhibit a divergent power-law behavior as *dM*/*dH* ~ *H*^−*α*^ in proximity to the BKT phase. At *T*_L_, *α* has the value of 2/3, which corresponds to a critical exponent *η* = 1/9 at the lower-BKT transition and is originated from the sixfold symmetry breaking^23^. The exponent *α* gradually decreases as temperature increases, and above an intermediate temperature between *T*_L_ and *T*_U_, *α* = 0 due to non-universal contributions. This is exactly what we observe in Fig. 3c. We fit the *dM*/*dH* with the power-law function at different temperatures, with the fitting regime chosen between 0.6–0.9 T. At 0.8 K, *α* is very close to the expected value of 2/3 (and thus *η* = 1/9) at *T*_L_, which constitutes a remarkable fingerprint evidence for the BKT transition. At lower temperatures such as 0.4 K, the exponent is also close to 2/3, because the susceptibility saturates with temperature, as shown in Fig. 3a. At high temperatures, *α* drops rapidly to a small value 0.12 at 2.1 K and becomes effectively zero at 3.0 K.

Therefore, the susceptibility scaling gives the lower-BKT transition at about 0.8 K and upper transition probably between 2.1 and 3 K, in good agreement with the *T*_L_ and *T*_U_ estimated from NMR. These results are also fully consistent with our QMC calculations on the susceptibility shown in Fig. 3d. At *T*_L_ or lower, *α* is 2/3, then decreases to a very small exponent 0.086 at 2.67 K, and above 3 K, becomes zero within numerical uncertainty. Such a power-law behavior in *dM*/*dH* again signifies the finite-temperature window of the BKT phase with diverging magnetic correlations, which gives rise to the universal power-law scaling of magnetic susceptibility.

## Discussion

We believe the findings in this work are of various fundamental values. Since the original proposal of a BKT phase in magnetic films^3–5^, which also triggered the currently thriving research field of topology in quantum materials, tremendous efforts have been devoted to finding the BKT phase in magnetic crystalline materials, yet hindered by the obstacle outlined in the Introduction. Here, benefiting from NMR as a sensitive low-energy probe, and the nearly zero planar gyromagnetic factor in a TLI magnet TmMgGaO_4_, we are able to reveal two BKT transitions and a critical BKT phase with an emergent *XY* symmetry. Together with the power-law behavior of the susceptibility and excellent agreement between our QMC simulation and experiment data, we unambiguously identify the long-sought BKT phase in a magnetic crystalline material.

Many intriguing questions are stimulated, based on the phase diagram in Fig. 1 obtained here. First, what is the nature of the ordered phase under finite fields, are there further exotic phases and transitions in the phase diagram, and will there be a field-induced quantum phase transition at the high-field side of the dome—these are all of great interests to be addressed in future studies. Second, it should be noted that the dynamical properties obtained by QMC calculations in Fig. 2 are computed on a large, while finite-size, 36 × 36 lattice, which already produces 1/*T*_1_ data in very good agreement with the experimental measurements. Such a great agreement is surprising, given the possible existence of randomness from Ma/Ga site mixing in the material TmMgGaO_4_^16^, and also the lattice disorder revealed by the large high-temperature second moment of the NMR spectra (Supplementary Note 4). Although the random distribution in intrinsic transverse fields and spin couplings does not seem to alter the low-temperature spin-ordered phase and the sharp spin excitation line shapes^18,19^, its intriguing effects on the finite-temperature phase diagram of TLI and also the compound TmMgGaO_4_ call for further studies.

Third, in the study of BKT transition in superfluid systems, it has been observed experimentally and understood theoretically that additional dissipations also appear above the transition temperature due to fluctuations of vortices^6^. Hence, the plateau of 1/*T*_1_ we observe may also cover regions slightly above the upper BKT transition temperature. We leave this subtlety to future numerical and experimental efforts. Lastly, in general terms, whether there are other rare-earth magnetic materials in the same family of TmMgGaO_4_ that, acquire similar 2D competing magnetic interactions from highly anisotropic gyromagnetic factor and unique triangular-lattice structures, and also exhibit the BKT physics, is quite intriguing and calls for future investigations. All these directions are ready to be explored from here.

## Methods

### Crystal growth and susceptibility measurements

Single crystals were grown by the optical-floating-zone method with an image furnace (IR Image Furnace G2, Quantum Design). The natural cleavage surface of the crystals is the *ab* plane, which allows us to align the field orientation within an error of 2^∘^. The dc susceptibility was measured in a PPMS VSM (Quantum Design) for temperatures above 2 K and in a He-3 MPMS (Quantum Design) for temperatures ranging from 0.4 to 2 K.

### NMR measurements

The ^69^Ga (*I* = 3/2, *γ* = 10.219 MHz/T) NMR spectra were collected with the standard spin-echo sequence, with frequency sweep by a 50 kHz step using a topping tuning circuit. The NMR hyperfine shift was obtained by calculating the change of the first moment of the spectra. The spin-lattice relaxation rate 1/^69^*T*_1_ was measured by the inversion-recovery method, where a *π*/2 pulse was used as the inversion pulse. The NMR data from 1.8 K and above were measured in a variable temperature insert, and the data from 1.8 K and below were measured in a dilution refrigerator. The weak NMR signal at low fields and the rapid decrease of ^69^*T*_2_ upon cooling (Supplementary Note 5) prevented us to measure the 1/^69^*T*_1_ for in-plane fields <3 T, with temperature below 1.8 K. Whereas for in-plane fields of 4 T and higher, the sample could not be held in position because of the large anisotropy in the *g*-factor and unavoidable sample misalignment (≲2^∘^). At *T* = 1.2 K, we did not find any change of 1/^69^*T*_1_ with two different radio frequency excitation levels (14 mT and 24 mT), and with different frequencies across the NMR line, within the error bar.

### Triangular-lattice Ising model

At zero field, the intralayer couplings in TmMgGaO_4_ can be described by the TLI Hamiltonian,

1 $$H={J}_{1}\sum _{\langle i,j\rangle }{S}_{i}^{z}{S}_{j}^{z}+{J}_{2}\sum _{\langle \langle i,j\rangle \rangle }{S}_{i}^{z}{S}_{j}^{z}+\sum _{i}\Delta {S}_{i}^{x},$$

where *J*_1_ and *J*_2_ are the superexchange interactions among Tm^3+^, 〈*i*, *j*〉 and 〈〈*i*, *j*〉〉 refer to the nearest neighbors and the next-nearest neighbors, respectively, and Δ is the energy splitting within the non-Kramers doublet imposed by the crystal field. We have shown that the parameter set *J*_1_ =  0.99 meV, *J*_2_/*J*_1_ ≈ 0.05 and Δ/*J*_1_ ≈ 0.54 reproduces the experimental results of the transition temperatures and the inelastic neutron scattering spectra^19^.

In the TLI model [Eq. (1)], we can define a complex field *ψ* as a combination of the Ising (*Z*) components ${m}_{{\rm{A}},{\rm{B}},{\rm{C}}}^{z}$ on three sublattices, i.e.,

2 $$\psi ={m}_{{\rm{A}}}^{z}+{{\rm{e}}}^{i2\pi /3}\ {m}_{{\rm{B}}}^{z}+{{\rm{e}}}^{i4\pi /3}\ {m}_{{\rm{C}}}^{z}.$$

Notably, *ψ* = ∣*ψ*∣e^*iθ*^ is a complex order parameter that represents the emergent *XY* degree of freedom relevant to the BKT physics in the TLI model.

### QMC calculations

QMC simulations were performed in the path integral in the ${S}_{i,\tau }^{z}$ basis with discretization in space and time. The lattice of *L* × *L* × *L*_τ_, where *L* = 36 and *L*_τ_ = *β*/Δ*τ* with Δ*τ* = 0.05 and *β* = 1/*T*, were simulated with both local and Wolff-cluster update schemes^30,31^. The 1/*T*_1_ results were obtained by first computing the dynamical spin–spin correlation function $\langle {S}_{i}^{z}(\tau ){S}_{j}^{z}(0)\rangle$ and then acquiring its real-frequency dependence *S*(**q**, *ω*) from the stochastic analytic continuation^32^. We then determined the 1/*T*_1_ either by summing the contributions close to momentum *K* or over the entire Brillouin zone, as discussed in the Fig. 2d of the main text,

3 $${T}_{1}^{-1}({\bf{q}})=\frac{1}{{L}^{2}}\sum _{{\bf{q}}}| {A}_{{\rm{hf}}}({\bf{q}}){| }^{2}S({\bf{q}},\omega \to 0),$$

where *A*_hf_(**q**) is the hyperfine coupling form factor (see Supplementary Note 2). Similar analyses have been successfully applied to the QMC computation of NMR 1/*T*_1_ for the spin-1/2 and spin-1 chains^33,34^.

## Supplementary information

Supplementary Information

Peer Review File

## Data Availability

The data that support the findings of this study are available from the corresponding authors upon reasonable request.

## Code availability

All numerical codes in this paper are available upon request to the corresponding authors.

## References

[1] Berezinskii VL (1971). Destruction of long-range order in one-dimensional and two-dimensional systems having a continuous symmetry group I. classical systems. JETP.

[2] Berezinskii VL (1972). Destruction of long-range order in one-dimensional and two-dimensional systems possessing a continuous symmetry group II. quantum systems. JETP.

[3] Kosterlitz JM, Thouless DJ (1972). Long range order and metastability in two dimensional solids and superfluids. (application of dislocation theory). J. Phys. C Solid State Phys..

[4] Kosterlitz JM, Thouless DJ (1973). Ordering, metastability and phase transitions in two-dimensional systems. J. Phys. C Solid State Phys..

[5] Kosterlitz JM (1974). The critical properties of the two-dimensional xy model. J. Phys. C Solid State Phys..

[6] Bishop DJ, Reppy JD (1978). Study of the superfluid transition in two-dimensional ^4^He films. Phys. Rev. Lett..

[7] Hebard AF, Fiory AT (1980). Evidence for the Kosterlitz-Thouless transition in thin superconducting aluminum films. Phys. Rev. Lett..

[8] Epstein K, Goldman AM, Kadin AM (1981). Vortex-antivortex pair dissociation in two-dimensional superconductors. Phys. Rev. Lett..

[9] Resnick DJ, Garland JC, Boyd JT, Shoemaker S, Newrock RS (1981). Kosterlitz-Thouless transition in proximity-coupled superconducting arrays. Phys. Rev. Lett..

[10] Heinrich M, Krug von Nidda H-A, Loidl A, Rogado N, Cava RJ (2003). Potential signature of a Kosterlitz-Thouless transition in BaNi_2_V_2_O_8_. Phys. Rev. Lett..

[11] Cuccoli A, Roscilde T, Vaia R, Verrucchi P (2003). Detection of *X**Y* behavior in weakly anisotropic quantum antiferromagnets on the square lattice. Phys. Rev. Lett..

[12] Wawrzy ńska E (2008). Charge disproportionation and collinear magnetic order in the frustrated triangular antiferromagnet AgNiO_2_. Phys. Rev. B.

[13] Wheeler EM (2009). Spin dynamics of the frustrated easy-axis triangular antiferromagnet 2*H*-AgNiO_2_ explored by inelastic neutron scattering. Phys. Rev. B.

[14] Tutsch U (2014). Evidence of a field-induced Berezinskii–Kosterlitz–Thouless scenario in a two-dimensional spin–dimer system. Nat. Commun..

[15] Kumar R (2019). Structural, thermodynamic, and local probe investigations of the honeycomb material Ag_3_LiMn_2_O_6_. Phys. Rev. B.

[16] Li Y (2020). Partial up-up-down order with the continuously distributed order parameter in the triangular antiferromagnet TmMgGaO_4_. Phys. Rev. X.

[17] Cevallos FA, Stolze K, Kong T, Cava RJ (2018). Anisotropic magnetic properties of the triangular plane lattice material TmMgGaO_4_. Mater. Res. Bull..

[18] Shen Y (2019). Intertwined dipolar and multipolar order in the triangular-lattice magnet TmMgGaO_4_. Nat. Commun..

[19] Li H (2020). Kosterlitz-Thouless melting of magnetic order in the triangular quantum Ising material TmMgGaO_4_. Nat. Commun..

[20] Moessner R, Sondhi SL (2001). Ising models of quantum frustration. Phys. Rev. B.

[21] Isakov SV, Moessner R (2003). Interplay of quantum and thermal fluctuations in a frustrated magnet. Phys. Rev. B.

[22] Liu, C., Huang, C.-J. & Chen, G. Intrinsic quantum Ising model with intertwined multipolarness on a triangular lattice magnet TmMgGaO_4_. *Phys. Rev. Research* **2**, 043013 (2020).

[23] Damle K (2015). Melting of three-sublattice order in easy-axis antiferromagnets on triangular and Kagome lattices. Phys. Rev. Lett..

[24] Biswas S, Damle K (2018). Singular ferromagnetic susceptibility of the transverse-field Ising antiferromagnet on the triangular lattice. Phys. Rev. B.

[25] Julien M-H (2000). ^63^Cu NMR evidence for enhanced antiferromagnetic correlations around Zn impurities in YBa_2_Cu_3_O_6.7_. Phys. Rev. Lett..

[26] Kitagawa K, Katayama N, Ohgushi K, Yoshida M, Takigawa M (2008). Commensurate itinerant antiferromagnetism in BaFe_2_As_2_: ^75^As-NMR studies on a self-flux grown single crystal. J. Phys. Soc. Jpn..

[27] Koutroulakis G (2015). Quantum phase diagram of the *S* = 1/2 triangular-lattice antiferromagnet Ba_3_CoSb_2_O_9_. Phys. Rev. B.

[28] Jeong M (2017). Magnetic-order crossover in coupled spin ladders. Phys. Rev. Lett..

[29] Moriya T, Ueda K (2003). Antiferromagnetic spin fluctuation and superconductivity. Rep. Prog. Phys..

[30] Wang Y-C, Qi Y, Chen S, Meng ZY (2017). Caution on emergent continuous symmetry: a Monte Carlo investigation of the transverse-field frustrated Ising model on the triangular and honeycomb lattices. Phys. Rev. B.

[31] Jiang, W., Pan, G. & Meng, Z. Y. Solving quantum rotor model with different Monte Carlo techniques. Preprint at https://arxiv.org/abs/1912.08229 (2019).

[32] Sandvik AW (2016). Constrained sampling method for analytic continuation. Phys. Rev. E.

[33] Sandvik AW (1995). NMR relaxation rates for the spin-1/2 Heisenberg chain. Phys. Rev. B.

[34] Capponi S, Dupont M, Sandvik AW, Sengupta P (2019). NMR relaxation in the spin-1 Heisenberg chain. Phys. Rev. B.

