# Supplementary material for: Evidence of the Berezinskii-Kosterlitz-Thouless phase in a frustrated magnet
Source: Nat Commun. 2020 Nov 6;11:5631. doi: 10.1038/s41467-020-19380-x (PMC7648753; doi:10.1038/s41467-020-19380-x)
Supplement: Supplementary file 1 — Supplementary Information [file 41467_2020_19380_MOESM1_ESM.pdf]

Supplementary Information for

Evidence of the Berezinskii-Kosterlitz-Thouless Phase in a Frustrated Magnet

Hu *et al*.

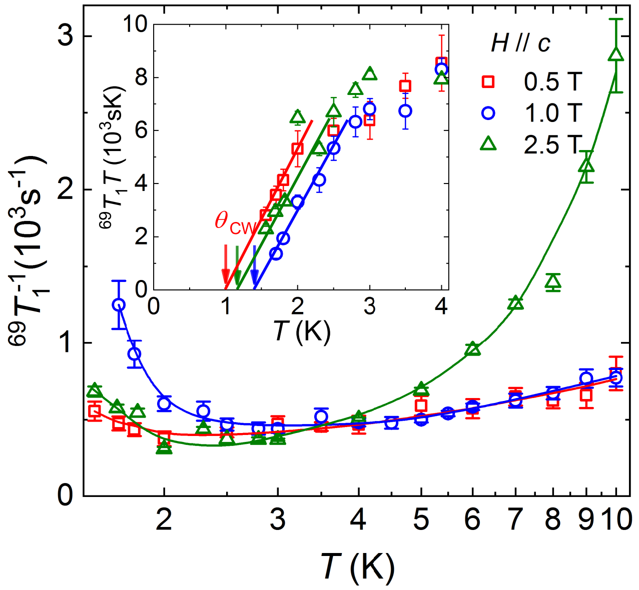

Supplementary Figure 1. **Spin-lattice relaxation rate** $1/^{69}T_1$ **under out-of-plane fields**. The $1/^{69}T_1$ data are shown as functions of temperatures at three fields, 0.5, 1, and 2.5 T, which are nearly identical at temperatures between 3 and 4 K, exhibiting similar paramagnetic spin fluctuations. Upon cooling, an upturn appears below 3 K, indicating the onset of low-energy magnetic fluctuations toward the magnetic ordering. The inset shows the $^{69}T_1T$ plotted against temperature, with a Curie-Weiss fitting below 2.5 K, which provides an estimated temperature $\theta_{\rm CW}$ (noted by down arrows) at which spin fluctuations are maximal. These temperatures are plotted in Fig. 1 in the main text.

Supplementary Note 1: Determination of the $\theta_{\rm CW}$ from the spin-lattice relaxation rate under out-of-plane fields

The spin-lattice relaxation rate $1/T_1$ quantifies the magnetic fluctuations through

$$\frac{1}{T_1} = T\sum_{\mathbf{q}} |A_{\rm hf}(\mathbf{q})|^2 \frac{{\rm Im}\chi(\mathbf{q},\omega)}{\omega}, \tag{1}$$

where $A_{\rm hf}$, ${\rm Im}\chi(\mathbf{q},\omega)$, and ω are the hyperfine coupling constant, the imaginary part of the dynamic spin susceptibility, and the NMR frequency, respectively.

The temperature dependence of $1/^{69}T_1$ were measured under various out-of-plane fields, and the data are shown in Supplementary Fig. 1. At fields $\mu_0 H = 0.5$ T and 1 T, $1/^{69}T_1$ shows a weak temperature dependence from 3 K to 10 K. However, for $\mu_0 H = 2.5$ T, $1/^{69}T_1$ increases quickly above 3 K, indicating strong field-induced fluctuations whose origin needs to be addressed in future studies.

Below 2 K, $1/^{69}T_1$ data in Supplementary Fig. 1 show an upturn for all measured fields, indicating the onset of low-energy spin fluctuations upon cooling. We fit the $1/T_1T$ with the Curie-Weiss function [2] $1/T_1T \sim 1/(T-\theta_{\rm CW})$ (see the inset of Supplementary Fig. 1), and provide an estimate of the transition

temperature $\theta_{\rm CW}$ where the relaxation rate $1/T_1$ diverges due to critical fluctuations. We then collect the estimated $\theta_{\rm CW}$ at various fields and plot them in Fig. 1 of the main text, which are in excellent agreement with the transition temperatures determined by other means. In particular, $\theta_{\rm CW}$ follows a dome-like shape with fields, in accordant to the peak positions of $C_{\rm m}/T$ and dM/dH (Fig. 1 of the main text). Note the Curie-Weiss form of the $1/T_1T$ has been widely observed in correlated materials. It was originally introduced by T. Moriya et al. [2] in studying quasi-2D itinerant antiferromagnets, where $1/T_1T \propto \chi(\boldsymbol{Q})$, with $\chi(\boldsymbol{Q}) \approx (T - T_{\rm N})$ being the dynamical susceptibility at the magnetic wave vector $\boldsymbol{Q}$.

Supplementary Note 2: Hyperfine coupling form factor

As a local probe, the NMR relaxation rate $1/T_1$ sums up the magnetic fluctuations from all momenta [see Eq. (1)], weighted by the hyperfine coupling form factor

$$|A_{\rm hf}(\mathbf{q})|^2 = |\sum_j \tilde{A}_{\rm hf} e^{i\mathbf{q}(r_j - r_i)}|^2, \tag{2}$$

where $\tilde{A}_{\rm hf}$ is the local hyperfine coupling constant, $r_{i(j)}$ labels the position of Ga(Tm) ions, and j is NN sites of site i (see Supplementary Fig. 2a). By assuming an isotropic, constant $\tilde{A}_{\rm hf} = 1$, we compute the form factor $|A_{\rm hf}(k_x, k_y)|^2 = 1 + 4\cos\frac{k_x}{2}(\cos\frac{k_x}{2} + \cos\frac{\sqrt{3}k_y}{2})$, and show the results in Supplementary Fig. 2b. From the results, we find that the K point has a very small form factor (actually zero for isotropic $\tilde{A}_{\rm hf}$) while the M point, on the contrary, corresponds to a larger form factor.

Supplementary Note 3: Contributions to $1/T_1$ from momenta other than the K point

To compare with experiments, we performed quantum Monte Carlo simulations of the $1/T_1$ (see Methods), considering contributions from magnetic fluctuations at typical momentum points M and K (see the inset of Supplementary Fig. 3). The calculated results are plotted in Supplementary Fig. 3 for comparison. Note that M and K are the magnetic ordering wave-vectors of the stripe and the clock antiferromagnetic phases, respectively [1]. From the M point contribution, $1/T_1$ is significant only at relatively high temperatures, which drops dramatically at 2 K and is negligible when cooled down to below 1.2 K. In contrast, the $1/T_1$ contributed from the K$'$ point is dramatically enhanced below 2 K and peaked at about 1 K, as discussed in the main text. The distinctive temperature dependence of the $1/T_1$ contributions from these two momentum points suggests a competing mechanism at low temperatures. This can be understood because the clock phase (fluctuations centred at K) wins over the stripe phase (fluctuations centred at M) at $T_{\rm L}$. From the comparisons between numerics and experiments, we conclude that the measured $1/^{69}T_1$ [Fig. 2(c) in the main text] is dominated by the fluctuations with momentum centred around K at low temperatures. The

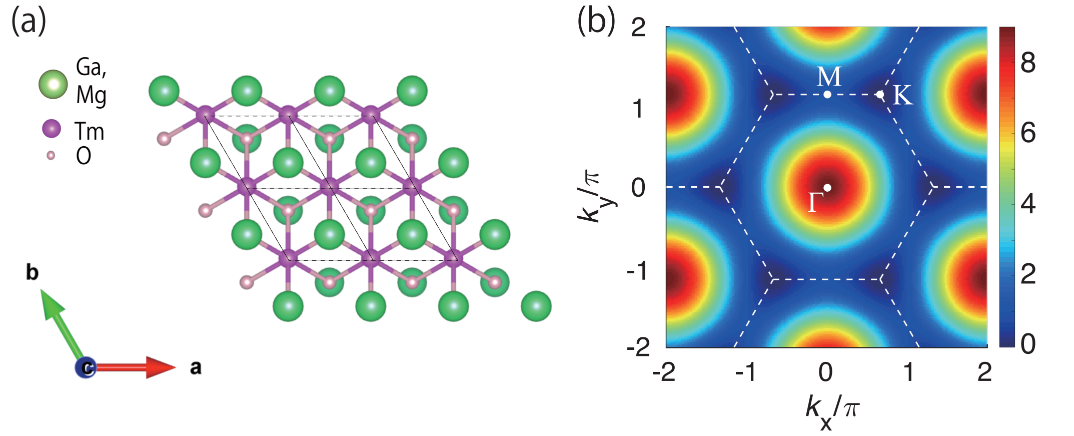

Supplementary Figure 2. (a) Lattice structure within the triangular plane where each Ga ion has three nearest Tm sites. (b) The contour plot of corresponding form factor $|A_{\rm hf}(\mathbf{q})|^{\mathbf{2}}$ considered in the main text.

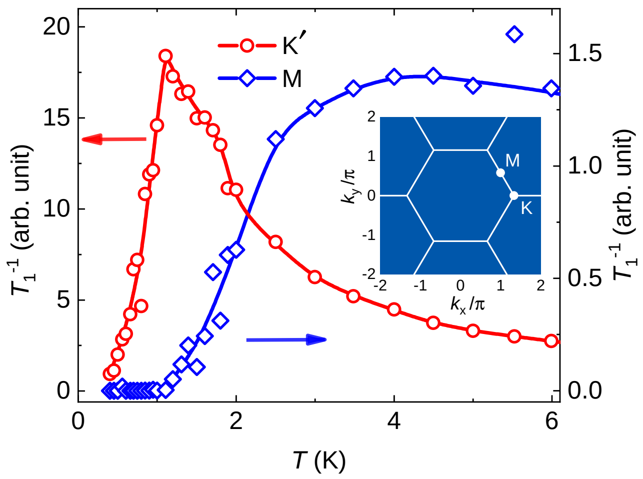

Supplementary Figure 3. The spin-lattice relaxation rate $1/T_1$ calculated by the quantum Monte Carlo simulations, including data measured at the M point (diamonds) and a vicinity point of K (circles), respectively. Inset illustrates the M and K points in the Brillouin zone of the reciprocal space.

$1/T_1$ contributions from M points, etc., start to play a role as temperature is escalated to above 2 K, due to the large density of states near the M point and the different hyperfine form factor $A_{\rm hf}(\mathbf{q})$, etc.

Supplementary Note 4: Second and third moments of the ^{69}Ga NMR spectra

The second and the third moments deduced from the ^{69}Ga NMR spectra, with 1 T and 3 T in-plane fields (i.e., $H//ab$), are plotted in the Supplementary Fig. 4. The second moment, defined as $\langle (f-\overline{f})^2\rangle^{1/2}$, which measures the standard deviation of the NMR spectra, roughly equals the full-width at half-maximum of the NMR peak. The second moment is about 0.85 MHz at T=10 K, much larger than the $1/^{69}T_2$ (4 kHz)

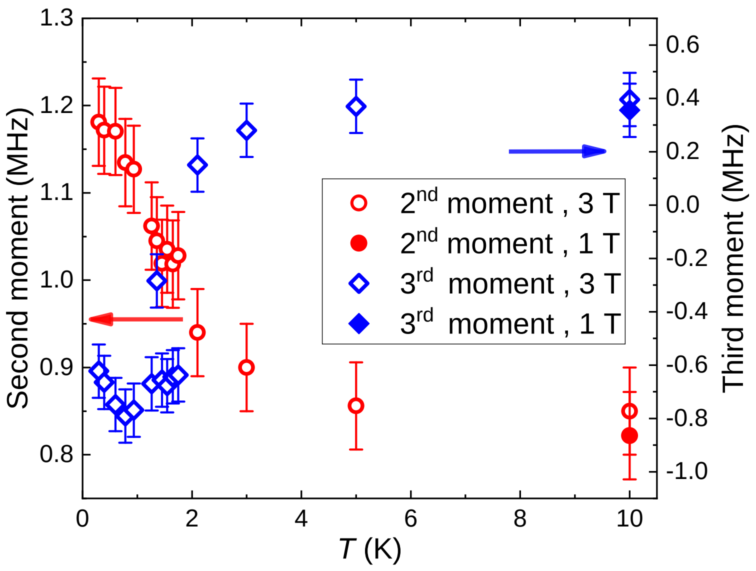

Supplementary Figure 4. The second and the third moment of the NMR spectra as functions of temperatures, under 1 T and 3 T field in the ab-plane.

under the same field (see Supplementary Fig. 5), indicating an inhomogeneous broadening of the spectra. At 5 K and above, the linewidth does not change much with temperature (cf. the data at 5 K and 10 K), nor with field (cf. the results under 1 T and 3 T), indicating that the high-temperature broadening does not correspond to a magnetic origin. Rather, since ^{69}Ga has a 3/2 nuclear spin, the width of the line is supposed to be defined by the unresolved quadrupolar splitting of the line (into three lines for one spin-3/2 nucleus), and therefore reflects the distribution of local electric-field-gradient (EFG)/quadrupolar couplings, which in principle barely changes with fields and temperatures. Therefore, the high-temperature broadening of the spectra indicates strong EFG inhomogeneity, mostly likely due to the Mg/Ga site mixing in the compound.

Below 2 K, on the other hand, the dramatic broadening of the NMR linewidth upon cooling indicates the the inhomogeneity of the local hyperfine field, as it first occurs when the quasi-static magnetic ordering develops below the BKT transition, and then in the magnetically ordered phase below 0.9 K. The frozen Mg/Ga site mixing, ought to also enhance this inhomogeneity, since the hyperfine field and/or the dipolar field produced by the Tm^{3+} magnetic ions on the ^{69}Ga nucleus is affected by its exact position and the neighbouring ions. However, it remains an open question to which extent the local moment of the ^{69}Ga, and consequently the magnetic properties, are affected by the site mixing. This is in particular thought-provoking, as the thermodynamic properties of $TmMgGaO_4$ can be well fitted by the TLI model without randomness, the magnetic order phase is maintained at low temperature, and the spin-wave dispersion remains sharp as observed in INS measurements.

The third moment, defined as $\langle (f - \overline{f})^3 \rangle^{1/3}$, measures the asymmetry of the NMR spectra. When

cooled below 2 K, the third moment undergoes a sign change from positive to negative (Supplementary Fig. 4), which actually reflects the shoulder-like feature developed to the left of the main peak of the spectra [Fig. 2(a) in the main text]. We ascribe this asymmetry of spectra to the dipolar hyperfine field with static/quasi-static ordering. However, to find out the accurate origin of these features and nail down the corresponding magnetic structures is beyond the scope of present NMR measurements.

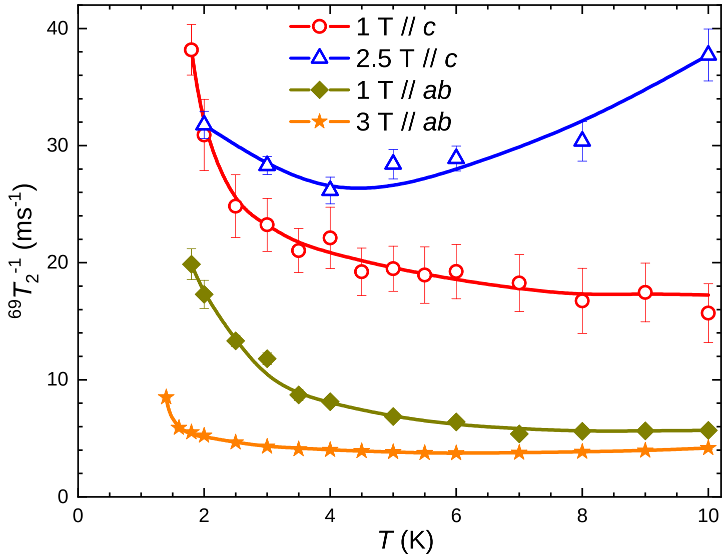

Supplementary Figure 5. The spin-spin relaxation rate $1/^{69}T_2$ as functions of temperatures under various in-plane (//ab) and out-of-plane (//c) fields.

Supplementary Note 5: Spin-spin relaxation rate $1/^{69}T_2$

The spin-spin relaxation rate $1/^{69}T_2$ with two field orientations are demonstrated in Supplementary Fig. 5. For the in-plane field $H//ab$, the $1/^{69}T_2$ data increase rapidly upon cooling below 3 K, which is consistent with the behaviours of $1/^{69}T_1$, indicating the onset of dominent contributions from the magnetic correlations. As the field increases from 1 T to 3 T, the $1/^{69}T_2$ data decrease (meaning longer spin-spin relaxation time). On the contrary, for the out-of-plane fields $H//c$, the $1/^{69}T_2$ data (above 2 K) increase when the field changes from 1 T to 2.5 T. These values are larger than those for the in-plane fields. In general, the spin-spin relaxation rate $1/T_2$ determines the decay rate of the NMR signal, and thus the spectra measurements become very challenging when $1/T_2$ is very large.

Supplementary Note 6: Magnetization curves at selected temperatures

The magnetization M of the sample was measured as functions of fields at various temperatures, as shown in Supplementary Fig. 6. The magnetization M increases with fields for all temperatures. At the

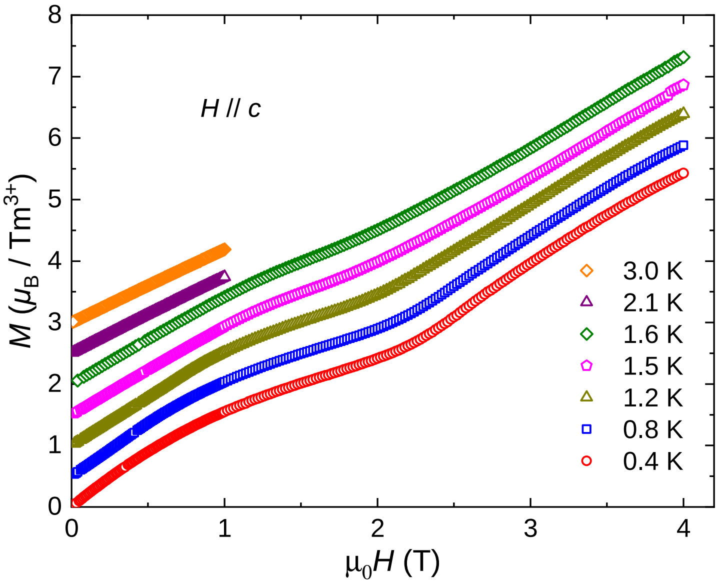

Supplementary Figure 6. The magnetization as functions of fields at selected temperatures. The measured data at different temperatures are shifted vertically for clarity.

lowest temperature 0.4 K, there are two inflection points at about $\mu_0 H = 1$ T and $\mu_0 H = 2.5$ T, respectively. The corresponding differential susceptibility dM/dH are shown and discussed in the main text.

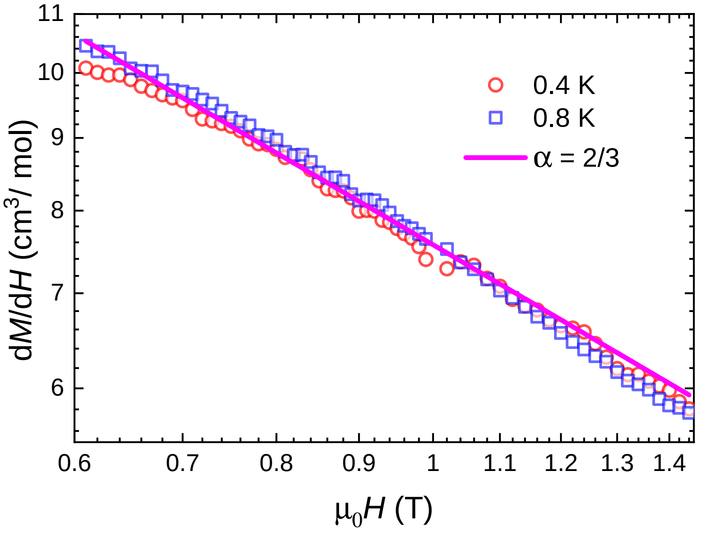

Supplementary Figure 7. Fittings of differential susceptibility at a larger field range. The solid line is the power-law function $dM/dH \sim T^{-2/3}$.

Supplementary Note 7: Differential susceptibility scalings

In both experimental and simulated data, we find only a limited parameter window to observe the power-

law scaling of differential susceptibilities. In Supplementary Fig. 7, we show that the scaling actually extends to a wider field range [0.6 T, 1.4 T], where we find the scaling works equally well. The fitted exponent $\alpha = 2/3$, in agreement with Fig. 3c in the main text, indeed satisfies the field-theoretical expectation and constitutes a strong evidence for the existence of BKT transition in the material $TmMgGaO_4$.

[1] Han Li, Yuan Da Liao, Bin-Bin Chen, Xu-Tao Zeng, Xian-Lei Sheng, Yang Qi, Zi Yang Meng, and Wei Li, "Kosterlitz-Thouless melting of magnetic order in the triangular quantum Ising material $TmMgGaO_4$," Nat. Commun. **11**, 1111 (2020).

[2] T. Moriya and K. Ueda, "Antiferromagnetic spin fluctuation and superconductivity," Rep. Prog. Phys. **66**, 1299–1341 (2003).
